# The Rise of Population Genomic Screening: Characteristics of Current Programs and the Need for Evidence Regarding Optimal Implementation

**DOI:** 10.3390/jpm12050692

**Published:** 2022-04-26

**Authors:** Kimberly S. Foss, Julianne M. O’Daniel, Jonathan S. Berg, Sabrina N. Powell, Rosemary Jean Cadigan, Kristine J. Kuczynski, Laura V. Milko, Katherine W. Saylor, Megan Roberts, Karen Weck, Gail E. Henderson

**Affiliations:** 1 Department of Genetics, University of North Carolina, Chapel Hill, NC 27599, USA; julianne_odaniel@med.unc.edu (J.M.O.); jonathan_berg@med.unc.edu (J.S.B.); sabrina_powell@med.unc.edu (S.N.P.); laura_milko@med.unc.edu (L.V.M.); karen.weck@unchealth.unc.edu (K.W.); 2 Department of Social Medicine, University of North Carolina, Chapel Hill, NC 27599, USA; jean_cadigan@med.unc.edu (R.J.C.); kriste.kuczynski@unc.edu (K.J.K.); gail_henderson@med.unc.edu (G.E.H.); 3 Department of Public Policy, University of North Carolina, Chapel Hill, NC 27599, USA; kwsaylor@pennmedicine.upenn.edu; 4 Division of Pharmaceutical Outcomes and Policy, Eshelman School of Pharmacy, University of North Carolina, Chapel Hill, NC 27599, USA; megan.roberts@unc.edu; 5 Department of Pathology and Laboratory Medicine, University of North Carolina, Chapel Hill, NC 27599, USA

**Keywords:** population screening, genomic testing, sequencing programs

## Abstract

Purpose: Advances in clinical genomic sequencing capabilities, including reduced costs and knowledge gains, have bolstered the consideration of genomic screening in healthy adult populations. Yet, little is known about the existing landscape of genomic screening programs in the United States. It can be difficult to find information on current implementation efforts and best practices, particularly in light of critical questions about equity, cost, and benefit. Methods: In 2020, we searched publicly available information on the Internet and the scientific literature to identify programs and collect information, including: setting, program funding, targeted population, test offered, and patient cost. Program representatives were contacted throughout 2020 and 2021 to clarify, update, and supplement the publicly available information. Results: Twelve programs were identified. Information was available on key program features, such as setting, genes tested, and target populations. Data on costs, outcomes, or long-term sustainability plans were not always available. Most programs offered testing at no or significantly reduced cost due to generous pilot funding, although the sustainability of these programs remains unknown. Gene testing lists were diverse, ranging from 11 genes (CDC tier 1 genes) to 59 genes (ACMG secondary findings list v.2) to broad exome and genome sequencing. This diversity presents challenges for harmonized data collection and assessment of program outcomes. Conclusions: Early programs are exploring the logistics and utility of population genomic screening in various settings. Coordinated efforts are needed to take advantage of data collected about uptake, infrastructure, and intervention outcomes to inform future research, evaluation, and program development.

## 1. Introduction

The past decade has witnessed a revolution in genomic sequencing capabilities, lowered costs of genetic testing, and expanded knowledge about gene-phenotype relationships. With these advances, consideration for genomic screening in the general population has garnered attention [1], and practice guidance has recently been published for screening in these populations [2]. Early implementation of such programs provides a landscape to learn about the clinical value, acceptability, implementation needs, and economic value of screening in general, unselected populations.

Health-information-seeking individuals have been able to gain some insight into health risk and trait associations through direct-to-consumer offerings such as 23andMe and others for more than a decade [3]. This testing typically includes polygenic risk scores, assessment of common polymorphisms, and carrier screening. In contrast, genetic testing for Mendelian conditions with a high risk for serious health outcomes has typically been restricted to patients who meet narrow clinical indications for testing. However, with the continued advancements in clinical genomics, there is great potential for health impact through population genomic screening for medically actionable, Mendelian conditions with a high risk for serious health outcomes and established medical guidelines for intervention.

In 2017, the Genomics and Population Health Action Collaborative (GPHAC) met to discuss and make recommendations for genomics-based screening programs for healthy adults [4]. The GPHAC chose to apply the CDC Office of Genomics and Precision Public Health’s suggested groupings:

Tier 1 (hereafter referred to as “CDC Tier 1 conditions”), genomic applications with a strong clinical knowledge base and strong evidence for medical actionability;

Tier 2, genomic applications with some evidence for medical actionability but not ready for routine implementation; and

Tier 3, genomic applications with early or limited evidence for possible medical actionability that may be candidates for further research.

The GPHAC Population Screening Working Group endorsed the 10 genes associated with the 3 CDC tier 1 conditions as being a reasonable starting point for primary screening in the general population, currently including Lynch syndrome (5 genes), hereditary breast and ovarian cancer (HBOC) (2 genes), and familial hypercholesterolemia (FH) (3 genes) [4]. The rationale for this recommendation is that these conditions are highly penetrant, with well-understood natural history and robust evidence-based clinical interventions to prevent or mitigate disease or disease risk in pre-symptomatic individuals, thus offering the greatest likelihood of maximizing benefit and minimizing harm. While the American College of Medical Genetics & Genomics (ACMG) Secondary Findings list was considered as a potential gene list for population screening, it was felt that not all genes included on this list had the same quality of supporting evidence and that certain characteristics, such as low penetrance or less comprehensive phenotypes, prevented them from inclusion in population screening [4]. As the genetic knowledge base increases, additional evidence will undoubtedly support integrating more genes into genomic screening programs [5] or perhaps removing genes from screening panels as appropriate.

To guide pilot screening programs, a set of 12 considerations were proffered by Murray and colleagues [1] (see Table 1), with the call for research to fill evidence gaps identified in these areas. These considerations highlighted the need to focus on principles underlying implementation choices. By 2021, recognizing that population screening programs were already taking place across the country, the ACMG published two sets of guidelines, one for organizations [2] and the second for individuals [6], outlining what should be considered when unselected populations are screened for highly actionable genes.

Early research, including our own pilot program, primarily aimed to understand why healthy individuals decide to participate in preventive genomic screening and what benefits and harms they might experience [7,8]. There is still little consolidated information about the current landscape of genomic screening in the U.S. In particular, it can be challenging to find and compare characteristics of early implementation programs, including who is screened, how participants are recruited, which genes are included, who pays for the screening, how results are disclosed, and how/if programs plan to expand over time. To address this gap, we collected information from current and/or recently concluded population genomic screening programs based on the categorical framework suggested by Murray and colleagues [1] (see Table 1). In this article, we present data on 12 preventive genomics programs in the U.S. that offer genomic screening. Our goal was to explore how programs are being implemented and how and why they vary across important characteristics and develop knowledge to guide future screening program development.

## 2. Materials and Methods

Genomic health screening programs were identified in a multistep process that included: broad Internet searches using combinations of terms, PubMed literature search, national conference presentations, and professional networks. Our goal was to reflect the landscape of population genomic screening programs offered in the United States. We recognize that our aim to identify and describe a representative sample was not a systematic survey and may have missed some such programs. Indeed, no census of genomic population screening programs exists.

Initially, a Google search was conducted in March 2020, that used varying combinations of the following search terms to identify possible programs: (population), (public), (genomic), (genetic), (screening), and (program). While this search returned with copious information, we focused specifically on genomic health screening programs that were available to the general population, not targeted to a specific disease group, gene-drug pair, or setting (e.g., employee wellness programs), or through direct-to-consumer programs or for-profit private companies.

To narrow our search, we excluded direct-to-consumer testing ordered without a medical provider, research-based programs including biobanks focused on particular diseases (e.g., cardiomyopathy), or programs that did not include a standard genomic screening analysis and returned results to patients. We also excluded genetic testing focused on individuals affected with a particular condition, including precision medicine efforts focused on cancer care and/or tumor sequencing. Due to complex health care access and financial coverage policies that are governed by individual country laws, as well as potential differences in international guidelines that may impact the availability of genomic screening in healthy populations, we chose to limit our search to United States-based programs.

A second, expanded search was conducted in September 2020 of web-based listings and scientific literature via PubMed, using the following terms (Gene/Genome/genomic Screen), (Preventative Genomics), (Precision Genomics), (Personalized Medicine), and (Precision Medicine). Of note, there were over 130 articles from PubMed that used the same search terms: (“Genetic Screen*”(ti) OR “genomic screen*”(ti) OR “genome screen*”(ti) OR “preventive genomic*”(ti) OR “precision genomics”(ti) OR “personalized medicine”(ti) OR “precision medicine”(ti)) AND (program*(ti) OR project*(ti)). The Sanford Chip program was later identified following their March 2021 publication “Precision Population Medicine in Primary Care: The Sanford Chip Experience”(9), and a final PubMed search was completed at that time to identify any new or additional programs.

To describe the features of each program, we employed 12 research/implementation questions identified by Murray and colleagues (see Table 1). These questions are framed as issues that a program should address when initiating population genetic/genomic health screening. For example, what population should be targeted with what genes and genomic technologies? How should the testing be paid for, including cascade testing and reanalysis? We used these questions as a guide for collecting information about each program and to characterize general trends across all the programs. In addition, we collected information on informed consent and access to genetic counseling. Primary information was collected based on publicly available sources from websites and publications. We then contacted program directors and/or other program staff to clarify, confirm, update and supplement initial data collection. Of note, we were not able to confirm all details with representatives from every program.

## 3. Results

**Figure 1:**
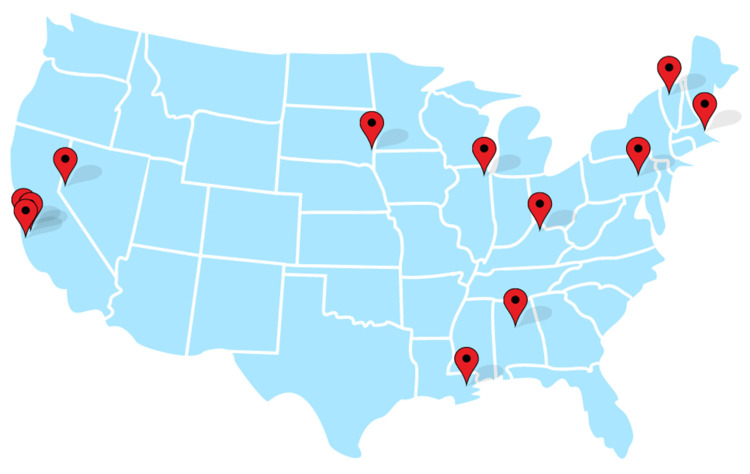
Locations of 12 screening programs.

We identified 12 programs that offer genomic screening to unselected populations in the U.S., listed in Table 2. They are widely dispersed geographically and located primarily at major tertiary care or academic medical centers, with several notable exceptions specifically targeting rural populations, described in more detail below (see map, Figure 1). All programs were associated with not-for-profit institutions, three public and nine private. We grouped them into four main categories: (a) health system-wide programs with primary care provider-based enrollment (*n* = 4); (b) clinical pilot projects inviting a pre-defined number of patients to participate (*n* = 3); (c) screening offered via statewide programs (*n* = 2); and (d) screening as an additional service in a genetics clinic (*n* = 3).

### 3.1. System-Wide Programs with Primary Care Provider Enrollment (n = 4)

- Geisinger Health System MyCode^®^—private, nonprofit health system

Geisinger, a large, integrated health care system in northcentral and northeastern Pennsylvania, partnered in 2007 with the Regeneron Genetics Center for DNA sequencing to offer the MyCode Community Health Initiative, a large research project coupling longitudinal electronic health data with a biobank and genomics data (exome sequence and genotype) [9]. Since its inception, there have been multiple clinical and research protocol changes, such as expanding eligibility to include children, changing the return of results protocols, and adjusting the list of genes sequenced. In 2013, the study protocol was amended to allow the return of genetic test results for medically actionable findings, including genetic variants associated with an increased risk for treatable and preventable heritable heart diseases and cancers [9]. These amendments allow for the inclusion of this program in our review. MyCode currently includes screening and return of results for the ACMG secondary findings list, version 2 (also known as ACMG SF v2), along with the *HFE* gene C282Y variant associated with hereditary hemochromatosis. A large staff assists with recruitment in outpatient clinics, and any positive results are returned by a genetic counselor. Follow-up with participants includes 6-week and 6-month post-disclosure contact, although these contacts seem to be associated with a separate research aim. MyCode is the largest program of its kind, building upon the robust clinical biobank protocol within its health research program. As of August 2021, the entire project contained 289,104 consented patient participants, with 202,280 samples received, 184,293 DNA sequences available for research, and 130,048 DNA sequences eligible and analyzed for clinical review, and 2682 clinical results reported (MyCode Scorecard). Working with Clear Genetics, Inc. (San Francisco, CA, USA). MyCode is attempting to develop scalable methods of returning results via “chatbots” to facilitate communication of results to family members for cascade testing [10]. Data are reanalyzed roughly every year.

- University of Vermont (UVM) Health Network “The Genomic DNA Test”—private, nonprofit health system

In 2019, the University Health Network in Burlington, Vermont, launched a pilot program with the goal of enrolling 1000 adult patients through UVM primary care practices by 2020 and 50,000 by 2025 [11]. They partnered with Invitae Laboratories for sequencing and LunaBPC for the storage of health data to be accessible for research projects. A total of 431 genes are analyzed, with customized action plans and care pathways for positive results given to a PCP. Genetic counselors are also available for the return of results and follow-up discussions. Additional offerings include free cascade testing for blood relatives and low-cost testing for partners related to carrier testing. This program is viewed as an extension of preventative care with no cost to patients. As of August 2021, 156 participants had completed testing.

- University of California at San Francisco (UCSF) “3D Health—Data, Discovery, Diversity”—public, nonprofit health system

UCSF partnered with PerkinElmer in 2020 with internal funding to support free genome sequencing for patients. The aim is to build a genomic database that is representative of their patient population and to advance precision medicine efforts. The program is advertised through social media, and an article was promoted by UCSF public relations on their website [12]. Enrollment occurs at a doctor’s visit or by phone, with the goal of enrolling 1000 participants. While whole-genome sequencing is being performed, gene analysis on the ACMG SF v2 list, along with ancestry, carrier status, and pharmacogenetic information, is being returned to the patient. This program has enrolled 450 patients and sequenced 220 individuals as of April 2021, with seven participants receiving positive ACMG SF v2 results.

- Sanford Health “The Sanford Chip DNA Test”—private, nonprofit health system

In 2018, Sanford Health launched an array-based screening chip that is performed in their internal Imagenetics (Internal Medicine and Genetics Initiative) laboratory [13]. Sanford Health is the nation’s largest nonprofit rural health care system, headquartered in Sioux Falls, South Dakota, with offices in North Dakota and Minnesota. This screening program is funded by an external donation. Genetic counselors work directly with primary care clinics to assist with the testing process. Patients complete consent online and receive approval from their PCP for testing, with a patient price of USD 49 and no charge to veterans. Screening includes 57 genes, similar to the ACMG SF v2 list, and pharmacogenetic information, with results being returned in the medical record to the primary care team. Medically actionable results are disclosed by a genetic counselor, and a clinical appointment with a genetic specialist is offered. The screen is an array-based genotyping assay and does not include full gene sequencing. As of March 2021, over 11,000 patients were enrolled, with 90% of patients receiving at least one pharmacogenomic variant and 1.5% of patients receiving a medically actionable result [13]. “Uninformative results” are returned at the discretion of the provider. Planned future directions include polygenic risk scores, additional pharmacogenetic information, and a larger number of medically actionable hereditary predisposition conditions.

### 3.2. Patients Invited to Pilot Projects (n = 3)

- NorthShore University HealthSystem “DNA10k”—private, nonprofit health system

NorthShore, in Evanston, Illinois, partnered with Color Health Inc. (Burlingame, CA, USA) to offer complimentary testing to 10,000 adult patients from April 2019 to January 2020 [14]. Patients were invited to participate through messages on the electronic medical record (EMR) prior to a regular healthcare visit. This pilot is complete, and this screening is now offered by the Neaman Center for Personalized Medicine at a price of USD 175 for patients. The program used educational videos and consent through an EMR patient portal called Northshore Connect. The screening included a 74 gene next-generation sequencing panel including pharmacogenetics, hereditary cancer, and cardiovascular risk and a low-pass whole-genome assay for “fun facts and traits”. Results were available to participants through both the EMR and a Color Health patient portal and discreetly incorporated into the EMR. Genetic counseling with a Color Health, Inc. genetic counselor was required before the release of positive results. Recommendations for follow-up were provided by a specific Northshore clinic. As of early 2020, 8792 patients had consented to have their blood drawn, and 5784 of those patients had results reported, with 462 (8%) patients having a total of 478 pathogenic variants [15].

- Ochsner Health System “innovationOchsner Population Genetic Screening program”—private, nonprofit health system

Ochsner Health System in New Orleans, Louisiana, partnered with Color Health, Inc. in 2019 to offer complimentary testing for the CDC tier 1 conditions to 1000 adult patients [16,17]. Digital recruitment and enrollment of patients are conducted via the MyOchsner patient portal. Testing is ordered by a network provider, and Color Health, Inc. sends the patient a testing kit for the collection of a DNA sample. The patient portal through the Epic EMR is used for education, consent, and results disclosure, which integrates the program’s emphasis on clinical decision support tools and robust provider education to incorporate genetic screening into routine practice. Screening results are stored in the patients’ EMR to enable access by Ochsner physicians for discussion with patients and to facilitate follow-up genetic counseling, which is available through Color Health, Inc. The program has completed its first phase of sequencing the CDC tier 1 conditions in 1000 patients. The second phase planned will expand to 3000–5000 patients and include pharmacogenetics.

- Stanford Health Care “Humanwide”—private, nonprofit health system

This small pilot program at Stanford Health took place from January to December 2018 [18]. No external partner is described. Fifty patients partnered with their primary care team for a comprehensive portrait of their health, including genetic testing, wearable devices, and wellness assessments [19]. Patient ages ranged from 24 to 86, with diverse ethnicity and baseline health concerns [20]. Genetic testing included CDC tier 1 conditions, with pharmacogenetics also offered. Both pre- and post-test genetic counseling was required, and multiple data outcomes were tracked. According to their website, Humanwide patients “Underwent genetic assessments and a pharmacogenomic screening, which evaluated their individual physiologic response to medications based on their genetic profile” [21]. Evaluation of the pilot focused on promoting a model of precision medicine and patient-centered primary care.

### 3.3. Statewide Programs (n = 2)

- “Healthy Nevada Project” Renown Health”—private, nonprofit healthcare network

In 2016, Renown Health System in Reno, Nevada, partnered with Genome Medical and Helix to offer complimentary genetic testing to adult Nevada residents with the goal of enrolling 250,000 individuals, with a focus on rural and minority populations. Funding was provided by the Renown Health Foundation and Nevada’s Knowledge Fund. In 2018, Healthy Nevada performed whole-exome-based sequencing and returned results for the three CDC tier 1 conditions, plus ancestry and nutrigenomics information. According to a 2020 published report on Healthy Nevada, among 26,906 participants who consented and underwent exome-based sequencing between April 2018 and July 2019, 1.33% had a pathogenic or likely pathogenic variant for Hereditary breast and ovarian cancer (HBOC), Lynch syndrome, (LS), and familial hypercholesterolemia (FH), and 90% of these positive results had not been previously identified for the impacted individuals [22]. By September 2020, 47,000 individuals had been sequenced with several hundred HBOC and FH results returned [23]. Positive results for these CDC tier 1 conditions are currently returned by a genetic counselor through Genome Medical. Cascade testing is encouraged for all positive results and provided for individuals who screen positive for FH and have children. Patients are periodically contacted for follow-up surveys.

- University of Alabama at Birmingham (UAB) and Tuskegee University “Alabama Genomic Health Initiative”—public, nonprofit health system

In 2017, UAB, in partnership with HudsonAlpha Institute for Biotechnology, received funding through the Alabama legislature to offer genetic screening for two cohorts of patients: a population cohort with a screening of the ACMG SF v2 list and an undiagnosed patient cohort with whole-exome sequencing. The population cohort participants received targeted genotyping to identify pathogenic and likely pathogenic variants for actionable conditions. The program was described in a 2021 publication as having 5369 patients enrolled by the end of 2019, including patients from all 67 counties in the state (24). Outreach efforts in underserved areas with “pop-up” enrollment clinics were successful in the enrollment of minority patients. Patients with positive results (1.5% of those sequenced) were contacted by a genetic counselor, while patients with negative results received a letter explaining these results and the limitations of a genotype test [24]. In 2020, the program modified the enrollment strategy to recruitment through family medicine and primary care clinics and began requiring participants to share results with their providers. Pharmacogenetic testing was also added. These modifications were made to integrate genomic medicine into primary care and engage physicians in personal and family health risk assessments, surveillance, and continued care.

### 3.4. Clinic-Based Programs (n = 3)

- Brigham and Women’s Hospital “Preventative Genomics Clinic”—private, nonprofit health system

In 2018, the Medical Genetics clinic at Brigham and Women’s Hospital in Boston, MA, began to offer preventive genomics screening with whole-genome sequencing and proactive panels, supported by clinical staff and some research funds. The Preventive Genomics Clinic uses the Laboratory for Molecular Medicine, which is operated by Partners HealthCare Personalized Medicine, which has close relations with the programs offering the screening. Genetics professionals review personal and family history to help decide the most informative clinical testing strategy for an individual patient. Examples of services offered are preventative genomic sequencing for healthy individuals, genetic risk assessment for people who are adopted or do not know their family history, and guidance for follow-up on direct-to-consumer testing. The program is open to adults and children when accompanied by a parent. Genetic testing is most often self-pay by the patient, as insurance companies are unlikely to cover testing costs if patients do not meet diagnostic test criteria.

- St. Elizabeth Healthcare “Precision Medicine and Genetics”—private, nonprofit healthcare system

Serving communities in Kentucky, Ohio, and Indiana, St. Elizabeth Healthcare offers clinic appointments with a genetic counselor to determine whether to conduct disease-specific testing, which may be covered by insurance, or a Proactive Gene Screen, which is paid for by the patient at the cost of USD 250–350. Currently, the program uses Invitae laboratory’s proactive genetic panels, which include panels related to cancer (61 genes), cardiovascular conditions (75 genes), pharmacogenetics (25 genes), and a ‘genetic health’ panel that includes cancer and cardiovascular screens plus additional conditions (147 genes). At one time, this clinic was focused on employee health, but it expanded to serve the entire healthcare system. Results are returned by phone by a genetic counselor, with further opportunities for follow-up appointments if needed.

- UCSF “Preventative Genomics Clinic”—public, nonprofit health system

UCSF partnered with external clinical labs and their in-house CLIA-certified labs to offer proactive gene panels, pharmacogenetics testing, and carrier screening. Clinic providers counsel patients at risk for a hereditary condition based on their personal or family history and, if appropriate, offer screening for preventative healthcare and family planning. Approximately 40 patients were seen in 2020 for preventative health consultations.

### 3.5. Program Characteristics

Table 3 presents the results for 13 program characteristics. They are grouped by the four program categories. Aside from the fact that the programs are all relatively new (at least nine have been developed since 2016), the table demonstrates substantial diversity, even within the four categories we developed. The three clinic-based programs are the most similar to each other, while the two state-run programs differ the most. The programs sponsored by the health care systems are quite different as well.

Table 4 presents the gene targets for the programs, illustrating considerable heterogeneity in what genes are being tested. They range from the 10 to 11 genes (depending on the inclusion of the *LDLRAP1* gene associated with autosomal recessive familial hypercholesterolemia) associated with the CDC tier 1 conditions to the 59 genes on the ACMG SF v2 list to broader groups of genes with less clinical relevance (e.g., ancestry testing), to whole-genome sequencing. Of note, some programs are built on the backbone of a broader population genomics research study and may perform whole-exome or genome sequencing with the return of only some results to participants (UCSF 3D). In these scenarios, the research aims may be broader and require research consent with an appropriate IRB overview. The most common panels are those based on the ACMG SF v2 list, which is used by six programs; the majority of these genes are also included in the proactive panels offered by the three clinic-based programs.

## 4. Discussion

As a team currently developing a genomic screening clinical offering, we were interested in information from other similar programs about who is screened, what genes are targeted, funding sources, and how population screening might be incorporated into primary care settings. Despite the small number of programs we identified, our analysis reveals sizable variation in their features. The 12 programs are almost equally split between academic and health care settings. The majority use commercial testing laboratories, perhaps because testing on a population scale may require the resources of a large commercial testing laboratory. Ten programs use sequencing, while two use a genotype array-based methodology. Array-based genotyping can streamline cost and variant interpretation but limits the reportable list of variants to a subset of “known” variants included in the array design. Gene analyses included in screenings range from a small number of actionable conditions, such as the CDC tier 1 conditions, to large panels that include pharmacogenomics, nutrigenomics, ancestry, and other “fun facts and traits”. The inclusion of cascade testing by nine of the programs suggests program efforts to maximize the potential for disease prevention and control, with the possible consideration of recouping some amount of initial economic investment on a population scale.

These are snapshots of relatively new and rapidly evolving programs. The two statewide programs, Healthy Nevada and Alabama Genomic Health Initiative, began in 2016 and 2017, respectively, and have recently published early program outcomes, as has Sanford Chip, which was established in 2018. Two of the pilot programs, Northshore’s DNA10k and Stanford’s Humanwide, are closed to recruitment, and the Ochsner program is in the second phase of its pilot. Northshore’s program has now been transferred to the Neaman Center for Personalized Medicine, and Stanford’s very small pilot became a model for precision primary care at their institution.

### 4.1. Economic Considerations

The budgetary impact of genetic screening programs and out-of-pocket costs for patients are key considerations for implementation, especially beyond initial pilots. Half of the programs included in this article are supported by institutional funding and offer population screening free of charge to individuals. One program mentioned self-pay rates after reaching an enrollment threshold, while the three clinical programs mentioned self-pay rates for proactive screening panels as insurance companies are unlikely to cover this type of testing at this time. Healthy Nevada, funded by Renown Health and Nevada Educational fund, offers free testing to all state residents. The Alabama Genomic Health Initiative has funds from the state legislature to enable free testing. Sanford Chip received a large external donation for their program, allowing them to charge USD 49 for screening and no charge for veterans. In light of this variability, it will be interesting to measure uptake across programs with and without testing charges covered, given that higher out-of-pocket costs are likely to have a negative impact on access to these programs, especially among patients with lower socioeconomic status. Economic evaluation studies have projected that it may be cost-effective for health systems to provide free or reduced-fee population genetic screening for certain highly actionable conditions, but assessing the long-term costs and benefits of expanded panels will depend on the cost and efficacy of downstream interventions. Additionally, the federal Beneficiary Inducement Statute may limit the ability of programs affiliated with health systems to offer low-cost or no-cost genomic screening to recipients of Medicare or Medicaid insurance, even if such screening would not be a covered benefit. Longitudinal studies assessing the clinical outcomes and economic impact of population genomic screening will be more challenging to complete and are not yet available for many programs due to their recent launch dates. Finally, cascade testing for a known familial pathogenic variant is often offered to at-risk family members by commercial screening companies at low or no cost, and the opportunity to ascertain additional family members with genetic risk increases the cost-effectiveness of genetic screening programs. Although it would require major practice shifts, if population screening became truly population-wide, it could supplant the need for cascade testing and eventually negate the need for screening future generations for predominantly heritable conditions when parental genotypes are known.

### 4.2. Engagement and Recruitment Strategies

Primary care providers are central to recruitment for seven programs, either at outpatient visits or by electronic message prior to a clinical appointment. Social media is used to promote one program (UCSF’s 3D Health), while completely digital enrollment is used by three others (Ochsner, Northshore, and Sanford). These strategies will certainly continue to be advantageous during the COVID-19 pandemic and the continued expansion of virtual health platforms. Three programs emphasize recruitment of underserved, rural, and minority populations: Alabama Genomic Health Initiative, Healthy Nevada Program, and Sanford Chip, which helps these population screening offerings represent all demographics. The three clinic-based programs did not specify recruitment mechanisms. Patient population restrictions seem to align with the primary funding sources and recruitment strategies. Lastly, only two programs (Geisinger MyCode and Brigham and Women’s Preventive Genomics Clinic) offer screening of children in addition to adults.

### 4.3. Clinical Implementation—Health Services

Ideally, these early genetic screening programs will inform structural needs for similar programs in the future. While workforce needs are essential to program implementation, this aspect of program structure was not consistently identified on program websites or in publications and may very well change over different phases and protocol changes of a program. Nevertheless, most programs describe a formal consent process, result disclosure, and customized action plans and/or care pathways for positive results that include defined roles of genetic counselors and PCPs in post-test discussions, either internally or through a commercial genetic testing laboratory. Increased demand for genetic counseling, in the face of professional shortages of genetic counselors and other genetics professionals, may require alternative approaches for most patients undergoing screening, especially those with non-concerning personal or family history and negative screening results. Geisinger proposed an innovative approach for consent, counseling, and result disclosure using a “chatbot” [10], but the success of this model is yet to be determined. Other approaches will need to be investigated as well. At least two programs mention that PCP/providers are supplied with specific care plans for positive results. Six programs mention recording results in electronic health records, which will help with continuity of care and results follow-up, although some programs allow patients to opt out of this facet. About half of the programs mention participant follow-up in certain intervals and re-examining results for changes in variant classification. Variant re-evaluation is not applicable to the two programs using array-based technologies. While the targeted genotyping array approach used by Alabama and Sanford is sensible from a cost perspective, it is unclear if limitations regarding the clinical sensitivity of this testing are being disclosed and are transparent to participants and providers, especially in the context of a negative or uninformative result.

Looking within the 12 programs we identified, it appears that some program features are not independent of each other. For example, those programs that partner with a genetics laboratory for sequencing (such as Color Health, Inc.) are likely to include access to educational materials and genetic counseling services, and in turn, the range of genes included in the screen may be associated with an available workforce to discuss frequently returned categories such as pharmacogenetics. In contrast, other features may be independent, such as dedicated funding, clinical staff availability, and the ability of the program to offer genetic testing “in-house”. Many programs also include pharmacogenetics, which may require review from a pharmacist with an understanding of these specific types of results or the development of clear action plans for primary care physicians to follow. The effectiveness of such information may depend on the ability of electronic health record systems to store genomic screening results and provide actionable decision support to providers at the appropriate time.

Most programs reported using a commercial laboratory for sequencing. We did not gather information from the programs on the details of their relationships with these commercial sequencing companies, nor did we ask whether information about commercial partners was included in informed consent documents for participants. In our own experience developing a population-based genomic screening program, the business models of commercial sequencing companies can be quite diverse. For example, companies may intend to use participants’ data for internal product development or profit from selling data to external commercial entities. Future research should explore the variety of partnership models between genomic health screening programs and commercial companies, as well as participants’ views on possible commercial use of their samples and data. Patients and providers may have different perspectives on the importance of these details. To build trust, population-based screening programs should be transparent with participants about their relationships with commercial sequencing companies.

### 4.4. Patient Outcomes

Murray and colleagues included several types of outcomes on their checklist for population genomic screening programs: (1) short-term clinical outcomes, such as correcting diagnostic misattribution, pre-symptomatic diagnosis of cancer or heart disease (also known as previvors); (2) long-term clinical outcomes, such as incomplete penetrance (especially when the rate of disease manifestation is lower than current disease knowledge suggests) [25]; and (3) assessment of best practices regarding negative screening result reporting, which is critically important to avoid false reassurance [1]. The main outcome reported by the 12 programs featured in this article is the rate of medically actionable conditions. Some of the programs indicate they are assessing the efficacy and effectiveness of population genomic screening at the individual patient and health service outcome levels, although due to the recent implementation of these programs, clinical outcomes are not yet available.

In a recent article, Alabama reported that comparisons between genotype findings and personal and family history indicated a lower penetrance of variants related to cardiovascular disease than previously expected in an unselected population [24]. In a 2020 article, the Healthy Nevada Program showed that population screening can identify individuals with a molecular diagnosis of the CDC tier 1 conditions that would not have been otherwise identified [22]. These publications suggest that the penetrance and prevalence of established genetic conditions may be altered by screening in the general population. MyCode featured the value for translational research derived from combined electronic health records and genomic databases [10]. Despite these important observations, as noted, most of the programs are too new for robust outcome analyses, and those publications that do exist are not yet comparing their results to other similar population screening program outcomes. Unfortunately, there is no central reporting platform for these data to be accessed and compared.

Another important aspect of patient outcomes is the interpretation of results. Only one of the programs (Alabama) mentions returning negative results. Understanding how participants and providers interpret their negative results from a screening program is just as important as understanding the interpretation of positive results. Inclusion of autosomal recessive conditions, such as *LDLRAP1* associated with autosomal recessive familial hypercholesterolemia or *MUTYH* associated with autosomal recessive familial adenomatous polyposis, on genetic screening panels also provides another layer of complexity for pre- and post-test counseling. A positive result for an autosomal recessive condition requires two pathogenic variants to be identified in trans (which is not generally possible to determine from singleton genetic analysis). In addition, due to Hardy–Weinberg proportions, identification of heterozygous carrier status will vastly outnumber detection of true homozygous or compound heterozygous affected individuals. Carrier screening was offered by many of the programs, so it is essential for participants to understand the difference between being affected with an autosomal recessive condition and being a heterozygous carrier for reproductive and family planning purposes. Thus, having informative pre-test educational materials and clear consent for these results, in addition to a clear policy on disclosure of carrier status, is a critical aspect of this screening and can greatly impact patient outcomes.

### 4.5. Health Screening

An analysis of existing population screening programs is particularly timely given the popularity of screening unselected populations for “actionable” conditions, although the topic of actionability is one that is debated in the genetics community. According to the most recent ACMG recommendations, identifying CDC tier 1 conditions in unselected populations would have “significant potential for positive impact on public health based on available, evidence-based guidelines” [2]. At the same time, it is noted that there is an incomplete understanding of what Wilson and Jungner referred to as the natural history of disease as “natural history involves ‘penetrance,’ the proportion of individuals with a given genomic risk who show evidence of the associated clinical problem; and ‘expressivity,’ the range of clinical manifestations associated with a specific genomic risk; and ‘age of onset’” [2,26].

The 2021 ACMG recommendations warn about the dangers of overestimating penetrance and expressivity. The secondary findings guidelines state that the list of genes is intended to guide secondary analysis of genomic data generated as part of diagnostic care and “do not constitute a primary health screening recommendation or strategy” [2]. While the overlap between the medically actionable secondary findings genes and medically actionable genetic screening panels is expected, caution is warranted. Penetrance and expressivity require a robust knowledge base prior to consideration of the widespread implementation of screening for a given condition, and the data needed to address the natural history of all the conditions on the ACMG v2 Secondary Findings list has yet to be generated by the programs we describe here. As mentioned above, the GPHAC Population Screening Working Group did determine that the natural history of Lynch syndrome, hereditary breast and ovarian cancer (HBOC), and familial hypercholesterolemia (FH) were well understood with robust evidence-based clinical interventions. Population screening has the ability to expand the knowledge of the natural history of disease for many conditions, though, as most genetic testing has been completed in individuals with a prior personal or family history of disease. Prior publications from genomic screening programs have reported enrollment bias toward those with a family history suggestive of hereditary disease [24], likely stemming from enhanced interest from these individuals. Therefore, attention will need to be given to the exploration of penetrance and variability for genetic conditions in unselected populations.

Incomplete penetrance and variable expressivity can be conceptualized as a genomics equivalent of “overdiagnosis”, in which an individual may be given a molecular diagnosis of a hereditary condition for which they may never develop symptoms [24]. This concept is a well-known phenomenon in health screening more broadly, particularly cancer screening in which an indolent lesion may be identified that would never develop into a life-threatening malignancy [26]. That being said, one aspect that differentiates genomic screening from other common health screening tests is the potential for other family members to be at substantial risk for the same inherited condition. Yet, thus far, most programs we identified have not provided information about outcomes beyond rates of positive findings. While not mentioned specifically in materials about the programs, it is likely that genetic counseling for return of positive results includes discussion about the limitations in knowledge about penetrance and expressivity, and thus the actual chance the particular health outcomes will manifest. Ongoing monitoring of outcomes for the screened individuals and their family members will be crucial in establishing a more complete evidence base for the widespread implementation of genomic screening.

### 4.6. Limitations

A comparison of such diverse programs is only a first step in understanding the landscape of genomic screening across the U.S., further limited by the fact that we only focused on a certain type of population screening. Additional programs have appeared (and will continue to appear) since our initial search, such as “In Our DNA SC”, a large-scale population genomics initiative at the Medical University of South Carolina, in collaboration with Helix [27]. We also acknowledge that there may be similar programs currently ongoing or previously offered that we failed to identify or that were excluded based on our narrow scope. Some variables were not available to our investigation but would be important in the evaluation of the barriers and facilitators to population screening and the success of a program. These include patient satisfaction with the screening process, other healthcare providers’ satisfaction with the program, patients’ understanding of a positive and negative result, healthcare dollars saved with a positive result (e.g., avoidance of cancer care or major cardiac event) or negative result, and the ability to recruit a specific population, such as the medically underserved. Due to the diversity in the design of the programs, we had limited ability to adequately assess many of the endpoints and outcomes. Other limitations include our inability to fact-check each publicly available detail associated with the programs and understanding that certain details of each program may change over time. Our research simply offers a snapshot of each program.

## 5. Conclusions

Early genomic population screening programs have begun to explore the logistics and potential health impact of this new screening modality in various health care settings. The variation in settings, target populations, clinical protocols, and genes screened allows for rich and diverse data, which are all necessary to generate an evidence base for future research to evaluate the health outcomes and cost of population genomic screening. Given the expected prevalence of Mendelian conditions in the general population, harmonized clinical data collection is needed to investigate the clinical validity and utility of a preventive genomic approach, in addition to the long-term cost and feasibility implications. It would be extremely beneficial if the outcomes of each program were shared in a central collection so that limitations and strengths can inform other organizations that are implementing clinical population genomic screening programs. In the future, features of population screening programs are likely to remain diverse, yet lessons learned can help newer programs avoid costly and time-consuming errors.

## Figures and Tables

**Table 1:** Recommended 12 questions to be addressed in pilot studies of general population screening used for this study.

Recommended Question *		Corresponding Category for This Study
1.	How should screening be designed to offer inclusive benefits for the whole population (with specific attention to the poor, as well as underrepresented racial and ethnic groups)?	Recruitment/Enrollment
2.	What are the appropriate population characteristics for screening (e.g., age, sex)?	Target Population/ Eligibility
3.	What is the optimal testing strategy/technology (e.g., exome sequencing, multigene panel, single-nucleotide polymorphism array)?	Target Genes
4.	What are the ideal lead institutions for carrying out DNA-based screening (e.g., health care provider organizations, departments of public health, for-profit companies)?	Institution/Setting
5.	How should DNA-based screening (primary screen) be paid for (e.g., government funding, private insurance, self-pay)?	Funding/Cost to Patients
6.	How should clinical follow-up (secondary screen) be paid for (e.g., government funding, private insurance, self-pay)?	Return of Results/Clinical Follow-up
7.	How often should data be reanalyzed (e.g., compared with evolving databases like ClinVar (updated annually))?	Data Reanalyzed
8.	What strategy should be pursued for cascade testing (e.g., should at-risk family members be automatically contacted by health system)?	Cascade Testing
9.	What are the short-term clinical outcomes (e.g., correcting diagnostic misattribution, pre-symptomatic diagnosis of cancer or heart disease)?	Outcomes
10.	What are the long-term clinical outcomes (e.g., nonpenetrance, overdiagnosis)?	Outcomes
11.	What are the best practices regarding negative screening result reporting (critically important to avoid false reassurance)?	Return of Results/Clinical Follow-up
12.	What are the clinical workforce needs related to delivering DNA-based results and clinical follow-up at population scale (i.e., how many medical geneticists, genetic counselors, specialists, others)?	Clinical Workforce

* Murray MF, Evans JP, Khoury MJ. DNA-based population screening: potential suitability and important knowledge gaps. *JAMA*. doi:10.1001/jama.2019.18640.

**Table 2:** Genomic Screening Programs in 4 Categories.

Program and Location	System-Wide Program| PCP Enrollment of Patients (*n* = 4)	System-Wide Program|Patients Invited to Pilot Project (*n* = 3)	Statewide Program (*n* = 2)	Screening Offered in a Genetics Clinic (*n* = 3)
Geisinger MyCode, Danville, PA	X			
University of Vermont The Genomic DNA Test, Burlington, VT	X			
University of California at San Francisco (UCSF) 3D Health, San Francisco, CA	X			
Sanford Health The Sanford Chip, Sioux Falls, SD	X			
Northshore DNA10K, Chicago, IL		X		
Oschner Health innovationOchsner Population Genomic Screening Program, New Orleans, LA		X		
Stanford University Humanwide, Palo Alto, CA		X		
Healthy Nevada Project, Renown Health			X	
Alabama Genomic Health Initiative, UAB Medicine			X	
Brigham & Women’s Hospital Preventive Genomics Clinic, Boston, MA				X
St. Elizabeth Healthcare Precision Medicine and Genetics, Edgewood, KY				X
UCSF Preventive Genomics Clinic, San Francisco, CA				X

Program summaries.

**Table 3:** Program Characteristics.

Program Name	Geisinger MyCode	UVM Genomic Population Healthpilot	UCSF 3D Health—Data, Discovery, Diversity	The Sanford Chip DNA Test	Northshore DNA10K	Ochsner Population Genomic Screening	Stanford Humanwide	Healthy Nevada	Alabama Genomic Health Initiative	Brigham Preventive Genomics Clinic	St Elizabeth Precision Medicine and Genetics	UCSF Preventive Genomics Clinic
Recruitment ongoing	Yes	Yes	Yes	Yes	No	Unknown	No	Yes	Yes	Yes	Yes	Yes
Enrollment initiated by	Provider	Provider	Patient and/or Provider	Patient and/orProvider	Provider	Provider	Provider	Patient and/or Provider	Patient	Patient	Patient	Patient and/or Provider
Enrollment goal	Unknown	1000	1000	Unknown	10,000	1000	50	250,000	10,000	N/A	N/A	N/A
Eligibility	HCS patients	HCS patients	HCS patients	HCS patients	HCS patients (limited number)	HCS patients (limited number)	HCS patients (limited number)	State residents	State residents	No known restrictions	No known restrictions	No known restrictions
Inclusion of children	Yes	No	No	No	No	No	No	No	No	Yes	No	No
Methodology	Sequencing	Sequencing	Sequencing	Genotype Array-Based	Sequencing	Sequencing	Sequencing	Sequencing	Genotype Array-Based	Sequencing	Sequencing	Sequencing
Sequencing partner	Commercial	Commercial	Commercial	Internal	Commercial	Commercial	Unknown	Commercial	Commercial	Internal	Commercial	Internal and Commercial
Institution	HCS	Academic	Academic	HCS	HCS	HCS	Academic	HCS	Academic	HCS	HCS	Academic
Cost to patient	No	No	No	Yes	No	No	No	No	No	Yes	Yes	Yes
Reanalysis of data	Yes	Yes	Yes	N/A	Unknown	Yes	Unknown	Unknown	N/A	Yes	Unknown	Yes
Cascade testing available	Yes	Yes	Yes	Yes	Unknown	Yes	Yes	Yes	No	Yes	Unknown	Yes
Results returned by	GC	PCP	GC	GC/PCP	Patient Portal	Patient Portal	Determined w/patient	GC	GC	GC	GC	GC
Year of launch	2013	2019	2020	2018	2019	2019	2018	2016	2017	2018	Unknown	Unknown

**Table 4:** Target Genes.

	Geisinger MyCode	UVM Genomic Population Health Pilot	UCSF 3D Health—Data, Discovery, Diversity	The Sanford Chip DNA Test *	Northshore DNA10K	Ochsner Population Genomic Screening	Stanford Humanwide	Healthy Nevada	Alabama Genomic Health Initiative *	Brigham Preventive Genomics Clinic	St. Elizabeth Precision Medicine and Genetics	UCSF Preventive Genomics Clinic
**Health Predispositions**												
CDC tier 1 only						x	x	x				
ACMG version 2 secondary findings	x	x	x	x	x				x			
Clinical lab proactive panel										x	x	x
WGS										x		
**Other**												
Carrier status		x	x									x
Pharmacogenetics			x	x	x		x				x	x
Traits/ancestry			x		x			x				

* Array-based testing.

## Data Availability

No new data were created or analyzed in this study. Data sharing is not applicable to this article.
