# Correction: Foss et al. The Rise of Population Genomic Screening: Characteristics of Current Programs and the Need for Evidence Regarding Optimal Implementation. *J. Pers. Med.* 2022, *12*, 692

**DOI:** 10.3390/jpm14030234

**Published:** 2024-02-22

**Authors:** Kimberly S. Foss, Julianne M. O’Daniel, Jonathan S. Berg, Sabrina N. Powell, Rosemary Jean Cadigan, Kristine J. Kuczynski, Laura V. Milko, Katherine W. Saylor, Megan Roberts, Karen Weck, Gail E. Henderson

**Affiliations:** 1 Department of Genetics, University of North Carolina, Chapel Hill, NC 27599, USA; julianne_odaniel@med.unc.edu (J.M.O.); jonathan_berg@med.unc.edu (J.S.B.); sabrina_powell@med.unc.edu (S.N.P.); laura_milko@med.unc.edu (L.V.M.); karen.weck@unchealth.unc.edu (K.W.); 2 Department of Social Medicine, University of North Carolina, Chapel Hill, NC 27599, USA; jean_cadigan@med.unc.edu (R.J.C.); kriste.kuczynski@unc.edu (K.J.K.); gail_henderson@med.unc.edu (G.E.H.); 3 Department of Public Policy, University of North Carolina, Chapel Hill, NC 27599, USA; kwsaylor@pennmedicine.upenn.edu; 4 Division of Pharmaceutical Outcomes and Policy, Eshelman School of Pharmacy, University of North Carolina, Chapel Hill, NC 27599, USA; megan.roberts@unc.edu; 5 Department of Pathology and Laboratory Medicine, University of North Carolina, Chapel Hill, NC 27599, USA

## Text Correction

There was an error in the original publication [1] “The Rise of Population Genomic Screening: Characteristics of Current Programs and the Need for Evidence Regarding Optimal Implementation”.

In the original publication, the authors erroneously reported that the UCSF “Preventative Genomics Clinic” partnered with Color Health, Inc., but it did not.

A correction has been made to Section 3.4.

The paragraph “UCSF partnered with Color Health, Inc. to offer population health screening with institutional support, insurance billing, and available self-pay rates. The current Color Health, Inc. ‘Extended’ test offering includes 74 genes related to cancer, cardiovascular disease, and pharmacogenetics. Clinic providers counsel patients at risk for a hereditary condition based on their personal or family history and, if appropriate, offer screening for preventative health care and family planning. Approximately 40 patients were seen in 2020 for preventative health consultations” was replaced with “UCSF partnered with external clinical labs and their in-house CLIA-certified labs to offer proactive gene panels, pharmacogenetics testing, and carrier screening. Clinic providers counsel patients at risk for a hereditary condition based on their personal or family history and, if appropriate, offer screening for preventative healthcare and family planning. Approximately 40 patients were seen in 2020 for preventative health consultations.”

The authors state that the scientific conclusions are unaffected. This correction was approved by the Academic Editor. The original publication has also been updated.

## References

[1] Foss K.S., O’Daniel J.M., Berg J.S., Powell S.N., Cadigan R.J., Kuczynski K.J., Milko L.V., Saylor K.W., Roberts M., Weck K. (2022). The Rise of Population Genomic Screening: Characteristics of Current Programs and the Need for Evidence Regarding Optimal Implementation. J. Pers. Med..

